# The Influence of Social Word Features on Early Word Learning in Autistic and Non-Autistic Children

**DOI:** 10.3390/bs16010026

**Published:** 2025-12-22

**Authors:** Fatema Mitu, Eileen Haebig

**Affiliations:** Department of Communication Sciences and Disorders, Louisiana State University, Baton Rouge, LA 70802, USA; fmitu1@lsu.edu

**Keywords:** social word ratings, early word learning, vocabulary size of acquisition (VSoA), autism

## Abstract

Early word learning is a critical milestone for children, yet autistic children often experience delays in language development. Social communication differences are a core feature of autism and may contribute to variability in learning experiences. Prior research has shown that word-level features such as iconicity, concreteness, and input frequency shape the timing of word learning, but less is known about the role of social word features. This study examined whether social word ratings predict when words tend to be acquired by autistic and non-autistic children. Social word ratings were examined as a predictor of word-level autistic and non-autistic acquisition normative data, while accounting for word input frequency. Regression analyses demonstrated that social ratings significantly predicted vocabulary acquisition, even after controlling for word frequency. Additional analyses demonstrated that socialness ratings continued to be a unique predictor of word acquisition when other affective features of words were included in the model (i.e., arousal and valence); this was also the case when iconicity and concreteness were included. Importantly, differences in group and interactions with social ratings and group were not statistically significant in any of the models. Lastly, the pattern of highly social words being acquired later in vocabulary development was strongest for nouns; the association was non-significant when examining verbs separately. Thus, in addition to previously studied word features like concreteness, imageability, and iconicity, social word features are predictive of vocabulary acquisition. These findings highlight an overlap in word features that influence learning in autistic and non-autistic children.

## 1. Introduction

Language skills are a key predictor of developmental outcomes across childhood and adulthood in autistic individuals. These early language skills are associated with later cognitive abilities, social development, and adaptive functioning (Anderson et al., 2009; Hedvall et al., 2015; LeGrand et al., 2021). While language delay is not a defining feature of autism, many autistic children also experience delays in language development compared to their non-autistic peers (Charman et al., 2003; Ellis Weismer & Kover, 2015; R. J. Luyster et al., 2008). On average, autistic children produce their first words around 23 months—nearly one year later than non-autistic children (Eigsti et al., 2011; Mayo et al., 2013). Because of the importance of early language learning, understanding the developmental course of vocabulary growth is particularly important for autistic children. The current study aims to examine the association between word-level acquisition and word features, with a primary focus on social features, using autistic and non-autistic acquisition normative data.

### 1.1. Expressive Vocabulary in Autistic Children

Studies have shown that spoken vocabulary development in young autistic children is qualitatively similar to that of typically developing children but follows a delayed trajectory (Charman et al., 2003; R. Luyster et al., 2007). However, areas of divergence in early word learning patterns have also been identified. For instance, Haebig et al. (2021) found that minimally speaking autistic children produce proportionally more verbs than typically developing toddlers with vocabularies under 25 words. Similarly, Jiménez et al. (2021) reported relatively more verbs in autistic children’s vocabularies during early points of vocabulary learning (e.g., vocabulary sizes between 1 and 100). These findings indicate that autistic children likely broadly attend to similar information in their learning environments relative to non-autistic children, but some differences in processing and learning are likely present given that vocabulary knowledge sometimes diverges from vocabulary patterns in non-autistic toddlers. To better understand the mechanisms underlying these differences, researchers have examined how word-level features, such as frequency, imageability, and iconicity, are associated with word learning.

### 1.2. Word Features

Word features such as imageability, concreteness, and iconicity have been linked to earlier word learning in both autistic and non-autistic children. Highly imageable words, such as concrete nouns are typically acquired earlier than less imageable words, such as abstract verbs in non-autistic children (Gentner & Boroditsky, 2001; Ma et al., 2009; McDonough et al., 2011). Building on this, Lin et al. (2022) examined whether imageability predicted noun and verb acquisition in autistic and non-autistic children. Lin et al. found that both autistic and non-autistic children’s first words were highly imageable, and this effect remained even after accounting for word frequency. Thus, children with small vocabulary sizes had higher average word imageability scores relative to children with large vocabulary sizes. This imageability effect was strongest for nouns, but still a relevant feature for explaining verb vocabulary size.

Similarly, concreteness, which refers to the degree to which a word denotes perceptible entities has also been linked to earlier word learning in typically developing children (Swingley & Humphrey, 2018). Notably, this concreteness effect has been documented in word learning patterns of children who speak languages other than English as well as in children who speak English (Braginsky et al., 2019; Verhagen & Van Stiphout, 2022). According to the dual coding theory (Paivio, 2013), concrete words are easier to learn because they can be represented both verbally and perceptually.

Iconicity references the relationship between form and meaning; highly iconic words have similarities between the word and the referent objects, images or events (Perry et al., 2015). For instance, sound effect words like “shhh” onomatopoeic words (e.g., moo) are rated to have high iconicity. Perry et al. (2015) examined the iconicity of 600 early-acquired English and Spanish words and found that words rated as more iconic were generally learned earlier by typically developing children. Across lexical categories, onomatopoeic words (e.g., moo) and interjections (e.g., ouch) were rated as most iconic, followed by adjectives (e.g., sticky), verbs (e.g., stop), nouns (e.g., jeans), and function words (e.g., here).

Word frequency is an important predictor of early word learning. Children often learn the words they hear most from caregivers. This pattern is found across languages and in both autistic and non-autistic children (Braginsky et al., 2019; Goodman et al., 2008; Lin et al., 2022). Furthermore, word frequency has been found to predict both expressive and receptive vocabulary skills (Swingley & Humphrey, 2018). As such, it has been important for researchers to control for word frequency when examining the association between lexical acquisition and other word features.

Researchers have also begun to examine socialness as a word feature. Socialness has been defined as how strongly words involve social roles, agents, or interactions with other people (Horvath et al., 2018b; Jiménez et al., 2021). For example, “sleeping” involves only one participant and therefore would be considered to have a low social rating, whereas “catch” requires two participants (a thrower and a catcher), making it more socially complex. 

This social rating reflects the number of people typically involved in the action, not whether the word carries a positive or negative social emotion. For instance, “fighting” also involves two people and would therefore receive a high social rating, even though it represents a negative interaction.

Horvath et al. (2018b) examined the relationship between verb socialness and age of acquisition by analyzing two-year-olds’ productive vocabularies from the MacArthur–Bates Communicative Development Inventories (CDI; Fenson et al., 2007; Marchman et al., 2023) They compared typically developing children and late talkers. Horvath et al. found that toddlers learned highly social verbs—verbs that had more event participants, such as “hug” or “throw”, later than less social verbs like “sleep”. They reasoned that verbs that involve more agents/individuals are more socially complex and thus more difficult to learn. In addition, Horvath et al. reported that late talkers’ verb vocabularies contained fewer highly social verbs than those of typically developing toddlers.

Building on this work, Jiménez et al. (2021) collected socialness ratings for verbs to explore early vocabulary development in young autistic children, late talking toddlers, and typically developing toddlers. Using a 10-point scale, they asked adult raters to judge the socialness of early-acquired verbs that appear on the CDI. Their rankings of socialness were specific to whether the word typically involves interactions with other people. Jiménez et al. (2021) found that all groups produced highly social verbs early in vocabulary development; however, in verb lexicons of 1 to 25 verbs, autistic children produced verbs that had lower socialness ratings relative to late talkers and typical talkers—though this effect size was small. Similarly, Haebig et al. (2021) examined verb production and reported that autistic children, with significant language delays (including minimally speaking autistic children), produced slightly fewer highly social verbs than typically developing peers, although group differences were not statistically significant (*p* = 0.15). Thus, social word features may contribute to group differences in early vocabulary development demonstrated by autistic and non-autistic children, but more information is needed.

The current study sought to further examine the association between social word features and word acquisition in autistic and non-autistic acquisition norms. Differences in processing social word features may be present for autistic children because children on the autism spectrum often experience difficulties with social communication, including both understanding social information and engaging in social interactions (American Psychiatric Association, 2013). These differences in social processing and engagement may lead to differences in the influence of social features of words on word learning between autistic and non-autistic children.

Diveica et al. (2023) developed a large database of socialness norms for 8388 English words, providing a broader and more inclusive tool for investigating how social word features shape vocabulary acquisition. They defined socialness broadly to include social behaviors and interactions, roles and institutions, personal and group characteristics, and social values. However, cognitive and neural models of semantic processing suggest that social concepts involve several partially dissociable components such as agency/agentively, intentionality, cooperation versus competition, and interpersonal roles (Spunt & Lieberman, 2013; Schaafsma et al., 2015). The Diveica et al. (2023) ratings provide an adult-derived index of socialness that does not distinguish among these components.

The Diveica et al. ratings were collected from adult participants, who judged each word on how socially relevant its meaning was, using a 7-point scale. Thus, highly socially relevant words—words with high socialness ratings—would include “a social characteristic of a person or group of people, a social behavior or interaction, a social role, a social space, a social institution or system, a social value or ideology, or any other socially relevant concept” (Diveica et al., 2023, p. 463). Analyses demonstrated high reliability and validity of the ratings. Importantly, these socialness ratings were only weakly correlated with affective features of words—namely arousal and valence extremity (*r*s < 0.25). Furthermore, the socialness ratings were negatively correlated with concreteness, though this association was weak. Diveica et al. also found that socialness ratings explained unique variance in performance on several lexical tasks. For example, high-socialness words such as “friend” or “teacher” were recognized more quickly and accurately in lexical decision tasks than low-socialness words such as “rock” or “table” (Diveica et al., 2023).

### 1.3. Current Study

While the Horvath et al. (2018b) study and studies conducted by Haebig and colleagues are interesting, they offer only a small glimpse into the association between social word features and vocabulary knowledge because they focused only on verbs. Furthermore, Haebig and colleagues were limited in their ability to examine this association because autistic word acquisition data were not available at that time. Until recently, word-level acquisition norms, such as age of acquisition (AoA), were only available for typically developing children or late talking toddlers. The traditional AoA variable cannot be derived for specific words that are produced by autistic children because autistic children show wide variability in the ages at which they begin to produce spoken language. This lack of word-level acquisition data for autistic children has caused researchers to use different approaches to examine word features, such as calculating the average imageability score across of the words within a child’s vocabulary and testing whether that average correlates with the child’s vocabulary size across a group of children (e.g., Lin et al., 2022).

To address the lack of usefulness of chronological age as an acquisition reference for autistic children, Haebig et al. (2025) proposed the use of Vocabulary Size of Acquisition (VSoA), which indexes the size of a child’s vocabulary as a reference point for language development rather than chronological age as a reference for development. VSoA represents the vocabulary size at which at least half of the children who have that vocabulary size are reported to produce a given word (Haebig et al., 2025). For example, a word like *‘bird’* might have a small VSoA (e.g., 40), meaning that when children know about 40 words, at least half of the children have been reported to produce *‘bird’*. In contrast, a more complex word like *‘because’* might have a much larger VSoA (e.g., 600) and it typically appears only once children know several hundred other words. This approach parallels AoA, which estimates the age at which half of children produce a word, but VSoA substitutes vocabulary size for age which makes the variable more appropriate for autistic children. Importantly, VSoA scores for non-autistic children have been found to correlate very highly with AoA.

The current study aimed to examine the potential influence of social word features on spoken vocabulary acquisition in autistic and non-autistic children by using the recently published social word ratings data published by Diveica et al. (2023) and the recently developed word-level and group-specific vocabulary acquisition variable (VSoA; Haebig et al., 2025). The current study provides a complementary extension to the previous research that examined social word features and vocabulary development by examining words that extend beyond the verb syntactic class (Haebig et al., 2021; Horvath et al., 2018b; Jiménez et al., 2021). Thus, our research questions were:

1. Do social ratings of words relate to the vocabulary size of acquisition (VSoA) in young children?
2. Does the relationship between social ratings and VSoA differ between autistic and non-autistic children?

## 2. Methods

Based on prior evidence linking word-level properties to early word learning, we can hypothesized that if a word has a higher socialness rating (i.e., it involves more social agents or roles), then it will be learned at a larger vocabulary size (higher VSoA), indicating that socially complex words tend to be acquired later in development (Horvath et al., 2018b). If autism is associated with differences in social communication and language learning mechanisms, then the relationship between socialness ratings and VSoA will differ between autistic and non-autistic children.

### 2.1. Measures

#### 2.1.1. Socialness Ratings

We collected socialness ratings from Diveica et al. (2023). In their study, 8388 English words were individually rated by at least 30 adult participants on a 1–7 scale based on how much each word involves social roles, agents, or interactions with other people (1 = not social, 7 = highly social). Higher ratings represent words that typically involve highly social behaviors, social values, and social characteristics of individuals (e.g., teacher, playground, cute, hug), while lower ratings represent non-social concepts (e.g., rock, tiger, sticky, sleep). We identified the words that also appeared in the CDI dataset (*N* = 170), which included nouns (*n* = 102), verbs (*n* = 40), adjectives (*n* = 25), function words (*n* = 1), and other word types (games/routines, routines, *n* = 2). These 170 overlapping words were included in our analyses.

#### 2.1.2. Vocabulary Size of Acquisition (VSoA)

Word-level VSoA estimates were taken from Haebig et al. (2025). This measure reflects the typical point in vocabulary development (i.e., the size of a child’s vocabulary) when a word tends to be learned. VSoA conceptually is similar to age of acquisition but indexes vocabulary size as a reference of language development rather than chronological age. VSoA values were available separately for autistic and non-autistic children, which allowed for direct comparisons of acquisition patterns across groups. For each word, we linked its VSoA values with its socialness rating and frequency value. In our regression models, VSoA served as the dependent variable, while independent variables were word level socialness ratings, the group variable (autistic vs. non-autistic), word frequency and the interaction between socialness ratings and group. VSoA data were available for all but one of the 170 CDI words with socialness ratings from Diveica et al. (2023); within the non-autistic VSoA data, a VSoA value was not available for the word “hate”; this word was excluded from the analyses, which caused a slight difference in the degrees of freedom between groups.

#### 2.1.3. Word Frequency

Word frequency refers to how often children are exposed to a particular word in their everyday language input. Words that occur more frequently in child-directed speech are generally learned earlier than less frequent words. In this study, frequency values were derived from the CHILDES corpus (MacWhinney, 2000), which contains transcripts of naturalistic interactions between caregivers and children. For each word, we used the number of times it appeared across these transcripts as an estimate of how often children hear that word. Because raw frequency counts are often highly skewed, all frequency values were log10 transformed before analysis.

#### 2.1.4. Affective Ratings (Valence and Arousal)

As will be explained below, additional exploratory analyses were conducted to carefully examine the unique association between the socialness word feature and vocabulary acquisition, even after controlling for other word features. Relevant to socialness ratings, we included an investigation of other affective word feature scores. Valence and arousal norms were taken from Warriner et al. (2013). Valence reflects the pleasantness of a word’s meaning (1 = very unpleasant, 9 = very pleasant, e.g., “sick” and “happy”), and arousal indicates the level of emotional activation associated with the word (1 = calm, 9 = exciting, e.g., “sleep” and “scared”).

#### 2.1.5. Concreteness and Iconicity

In addition to including exploratory analyses that controlled for other affective features, we also explored the association between socialness and word acquisition after controlling for concreteness and iconicity. Concreteness ratings were obtained from Brysbaert et al. (2014), in which over 30,000 English words were rated on a 5-point scale (1 = very abstract, 5 = very concrete; e.g., “happy” and “apple”). Higher scores indicate that the concept can be more easily experienced through the senses. Iconicity values were collected from Perry et al. (2015), who collected adult ratings for 600 early-acquired words on how much a word’s form sounds like its meaning (1 = not iconic, 7 = highly iconic).

#### 2.1.6. Argument Structure

As previously noted, Horvath et al. (2018b) suggested that verbs that with multiple event participants may be learned later in development because they tend to appear in sentences that have more complex argument structure (i.e., intransitive verbs in sentences and transitive verbs in sentences). As such, we used Horvath et al.’s verb characteristics table to classify our 40 verbs as intransitive and transitive. Given that the vast majority of the verbs were classified as transitive (38 out of the 40 words), we also documented whether could be classified as intransitive or transitive (*n* = 17), depending on the sentential context. In addition, we documented whether the verbs could be classified as ditransitive (11 of the 40 verbs; Horvath et al., 2018b). This allowed us to examine whether socialness features were associated with transitivity classification.

### 2.2. Data Analysis Plan

All analyses were conducted in R (Version 4.5.1, R Core Team, 2023). We first calculated Pearson correlations between word-level socialness ratings and VSoA separately for the autistic and non-autistic groups. To evaluate whether socialness ratings overlapped with other lexical properties, we also ran Pearson correlations between socialness ratings and iconicity, concreteness, valence, and arousal. To address the possibility that socialness ratings might reflect argument-structure for verbs, we used *t*-test analyses to we compare socialness ratings between transitive verbs and verbs that could be transitive or intransitive and to compare socialness ratings between ditransitive verbs and verbs that could be transitive or intransitive. Next, we ran a multiple linear regression model predicting VSoA with the following independent variables: word-level socialness ratings, group (autistic vs. non-autistic), and their interaction, while controlling for word frequency. To evaluate whether a socialness effect persists after controlling for other lexical properties, we further extended our model by adding iconicity and concreteness, valence and arousal. Lastly, to determine whether socialness effects differed by syntactic class, we conducted separate analyses for nouns and verbs.

### 2.3. Data Availability

All datasets used in this study are publicly available. Socialness ratings are available from Diveica et al. (2023), VSoA data are available from Haebig et al. (2025), and word frequency counts are available from the CHILDES corpus (MacWhinney, 2000). As noted in the Method section, concreteness, iconicity, arousal, and valence scores are also publicly available (Brysbaert et al., 2014; Perry et al., 2015; Warriner et al., 2013, respectively).

### 2.4. Ethical Considerations

This study used publicly available, de-identified datasets and did not involve direct interaction with human participants. Therefore, informed consent was not required. The current study was approved by the Louisiana State University’s institutional review board (IRB).

### 2.5. Generative AI Statement

Generative artificial intelligence (GenAI) was not used to create this manuscript or to conduct analyses for the current study.

## 3. Results

### 3.1. Correlations Between Socialness and VSoA

**Figure 1:**
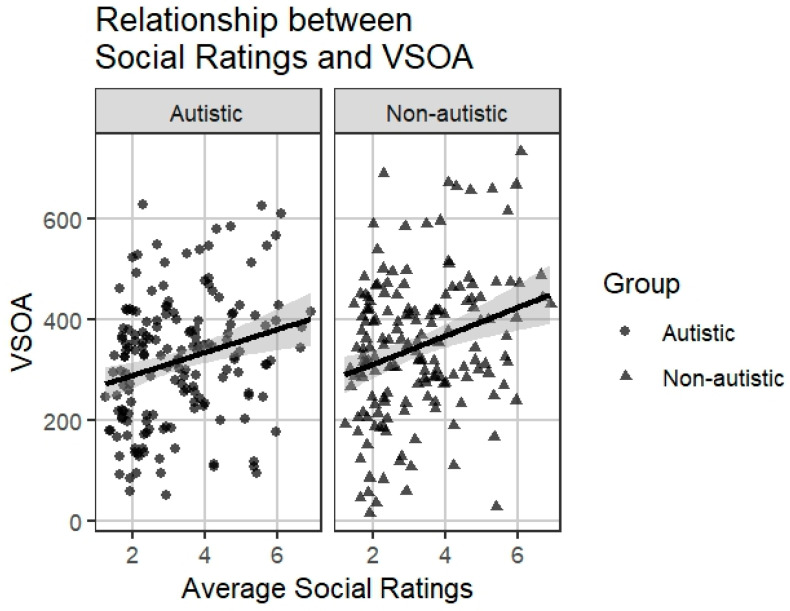
Relationship between Socialness Ratings and VSoA by Group. Note. Each point represents a word. The regression lines indicate the positive association between social ratings and group-specific VSoA value.

We first examined the bivariate associations between word-level socialness ratings and VSoA for autistic and non-autistic children. There was a moderate positive correlation between word-level socialness ratings and non-autistic VSoA values (*r* = 0.37, *p* < 0.001; *n* = 169), indicating that words with higher social content tended to be acquired at larger vocabulary sizes (i.e., later in vocabulary development). A similar moderate positive correlation was found between word-level socialness ratings and autistic VSoA values (*r* = 0.34, *p* < 0.001; *n* = 170). See Figure 1.

### 3.2. Bivariate Correlation Among Lexical Properties

To evaluate whether ‘socialness’ overlaps with other word level properties, we conducted additional Pearson correlations. Socialness was positively correlated with arousal, *r*(168) = 0.31, *p* < 0.001, indicating that more social words tended to be more emotionally activating. In contrast, socialness was not correlated with valence, *r*(168) = 0.07, *p* = 0.384. Furthermore, there were no significant correlations between socialness scores and concreteness *r*(168) = −0.08, *p* = 0.30 or between socialness and iconicity, *r*(153) = 0.14, *p* = 0.07. These patterns indicate that the association between socialness and VSoA is not explained by other lexical or affective properties; however, we further examine this using linear regression analyses.

### 3.3. Socialness and Argument-Structure Complexity

As socialness may also relate to grammatical complexity in verbs, we next examined whether socialness rating scores differed between transitive verbs (*n* = 21) and verbs that could be classified as transitive or intransitive or only as intransitive (*n* = 19). Socialness ratings did not statistically differ (*p* = 0.685). We also conducted a *t*-test to compare socialness rating scores for verbs that could be transitive or intransitive (*n* = 27) with socialness ratings for verbs that are ditransitive (*n* = 11); socialness ratings did not differ (*p* = 0.647). Thus, verbs that involved more complex argument structure (e.g., ditransitive verbs, e.g., show, give) did not systematically receive higher socialness ratings than verbs that can to serve as intransitive verbs, with simpler argument structure (e.g., wait, wish). This indicates that socialness ratings do not simply reflect argument-structure complexity.

### 3.4. Primary Regression Model: Predicting VSoA from Socialness, Group, and Frequency

Next, to address our primary research aim, we conducted a multiple regression analysis including socialness ratings, group (autistic vs. non-autistic), word frequency, and the interaction between socialness ratings and group. The overall model was significant, *F*(4, 334) = 24.79, *p* < 0.001, and explained approximately 22% of the variance in VSoA. Socialness ratings were a significant positive predictor of VSoA (B = 29.95, SE = 6.63, *t* = 4.51, *p* < 0.001), indicating that words with higher social content were learned later in vocabulary development. Word frequency was significantly and negatively associated with VSoA (B = −86.77, SE = 10.81, *t* = −8.03, *p* < 0.001), which suggests that more frequent words were learned earlier in vocabulary development. Group (autistic vs. non-autistic) was not a significant predictor nor was the interaction between group and socialness ratings (see Table 1). These results indicate that socialness word features are associated with when words tend to be learned even after accounting for word frequency, and importantly, that the relationship between socialness and VSoA did not differ significantly by group.

In the following sections, we present exploratory analyses that were conducted to examine whether socialness word ratings maintained their unique predictive value after controlling for other social and non-social word features.

### 3.5. Control for Affective Meaning

Given that socialness as a concept could encompass many subcategories of concepts, we conducted additional analyses to examine the unique contribution of Diveica and colleagues’ socialness ratings while also including social affective word features—arousal and valence. This expanded model explained significant variance in autistic and non-autistic VSoA data (*F*(333) = 8.26, *p* < 0.001; *R*^2^ = 0.11, adjusted *R*^2^ = 0.10). Socialness remained a positive predictor (B = 24.12, SE *=* 7.28, *t* = 3.31, *p* = 0.001). Valence was negatively associated with VSoA (B = −20.59, SE *=* 6.13, *t* = −3.36, *p* = 0.001), whereas arousal and interaction between socialness ratings and group were not statistically significant. Table 2 displays the full model findings.

### 3.6. Socialness Effects After Controlling for Other Lexical Variables

We next evaluated whether the socialness ratings effect persisted after adding lexical covariates in addition to the affective features. An extended multiple regression model including socialness ratings, group, word input frequency, iconicity, concreteness, affect, and valence, as well as the interaction between socialness ratings and group explained significant variance in word VSoA data. Socialness ratings remained a significant unique positive predictor of VSoA, B = 34.63, SE = 6.82, *t*(300) = 5.08, *p* < 0.001. Additionally, word frequency continued to predict word acquisition, as did valence. Iconicity also contributed significant unique variance in acquisition, B = –2.54, SE = 0.63, *t*(300) = –4.01, *p* = 0.001; highly iconic words are learned early in development. In contrast, concreteness was not a significant unique predictor. As previously reported, neither group nor the interaction between socialness ratings and group were significant. Table 3 provides the full model output.

### 3.7. Socialness Effects for Nouns and Verbs

Lastly, given that word features sometimes differentially predict word acquisition of different lexical classes (e.g., imageability, Lin et al., 2022), we examined the association between socialness and word acquisition separately for nouns and verbs. We first examined noun acquisition norms. Socialness ratings of nouns significantly predicted VSoA; nouns rated to be highly social tend to be acquired at later stages of vocabulary acquisition, B = 22.35, SE = 8.75, *t*(200) = 2.55, *p* = 0.01. This result indicates that more social nouns are acquired later. Neither group or interaction between group and socialness ratings was significant in predicting noun acquisition data. When valence and arousal were added, socialness remained a significant predictor, B = 26.44, SE = 9.02, *t*(198) = 2.93, *p* = 0.004, whereas valence and arousal were not significant.

After adding lexical properties iconicity, concreteness, and word frequency, the socialness effect remained robust (B = 28.52, SE = 6.44, *t*(169) = 4.43, *p* < 0.001). Concreteness strongly predicted noun acquisition, whereas iconicity was not significant. No group differences or interactions were observed. Table 4 presents the full noun model output.

A re-analysis of the primary regression model for our verbs revealed that socialness did not significantly predict verb VSoA (B = 7.36, SE = 16.53, *t* = 0.46, *p* = 0.65), and neither group nor the interaction between group and socialness was significant. After adding valence and arousal, pattern did not change. Then we extended our base model adding the lexical properties of iconicity, concreteness, and word frequency. We found that socialness remained nonsignificant, B = −4.61, SE = 11.71, *t*(72) = −0.39, *p* = 0.70. In contrast, unique explained variance in verb learning was yielded by verb input frequency (*B* = −90.80, *SE* = 17.70, *t* = −5.13, *p* < 0.001), concreteness (B = −79.38, SE = 14.41, *t* = −5.51, *p* < 0.001), and iconicity (B = −2.73, SE = 0.90, *t* = −3.03, *p* = 0.003). As in previous analyses, group and the interaction between group and socialness were not significant. See Table 5 for full model findings.

## 4. Discussion

The current study examined how socialness features of words is associated with when autistic and non-autistic children tend to acquire words. We used a newly developed word-level acquisition variable—Vocabulary Size of Acquisition (VSoA)—to more precisely examine this association with newly released social rating values (Diveica et al., 2023). We demonstrated that an under-examined word feature—socialness—is positively associated with word acquisition. Importantly, this association persisted after controlling for word frequency, concreteness, iconicity, and affective features (valence and arousal). However, socialness ratings did not significantly predict verb acquisition order. Furthermore, the association between socialness and word acquisition data did not differ between our autistic and non-autistic data, indicating an overlap in language processing and learning.

### 4.1. Socialness and Vocabulary Size of Acquisition (VSoA)

Our results demonstrated that words with higher socialness ratings were learned later in vocabulary development. This indicates that socially complex words, those involve multiple agents, roles, or high social characteristics or values are harder for children to learn early. These findings are somewhat consistent with previous studies on social complexity and word learning. Horvath et al. (2018b) showed that toddlers learned verbs that involve more participants (i.e., high socialness) later than verbs with only one participant. Horvath et al. (2018b) proposed that event complexity impacts processing in toddlers, with added challenges to identifying the salient individual for actions when multiple event participants are involved with a concept. Notably, Horvath et al.’s definition of event participants could also include instruments (e.g., eat—2 event participants). Thus, this reasoning about processing and learning may also extend to nouns (e.g., food being a more social word than tiger). In the current study, socialness was positively associated with VSoA across word classes, but when the analyses were restricted to verbs only, socialness—as defined by Diveica et al. (2023)—was not a significant predictor. This discrepancy may be due to the fact that Diveica et al. characterized socialness more broadly than Horvath et al. (2018b).

The current finding of no group differences aligned with Haebig et al. (2021), who did not observe statistical differences in verb social ratings between minimally speaking autistic children and typically developing toddlers. However, the current study’s findings contrast with Jiménez et al.’s (2021) findings. Jiménez et al. found that the early verb lexicons (1–25 verbs) that typically talking toddlers produced had higher average social ratings relative to larger verb vocabulary sizes (25–50 verbs within the lexicon); however, this effect was small (Cohen’s *d* = 0.11) and this difference in socialness scores between vocabulary sizes was not significant in autistic children and late talkers. Additionally, Jiménez et al. (2021) found that autistic children’s early verb vocabularies (1–25 verbs) consisted of verbs that were on average lower in socialness ratings relative to those of typically talking toddlers (small effect) and late talking toddlers (medium effect). Given that this small effect of socialness in verb vocabularies between the typical talkers and autistic children was observed only for children with verb vocabulary sizes between 1 and 25 verbs, this effect may not have held if verb vocabulary size was examined on a continuous scale.

The current study’s examination of socialness extends the previous child literature (Haebig et al., 2021; Horvath et al., 2018b; Jiménez et al., 2021) by studying socialness in a larger number of words and in words that span different syntactic classes. Additionally, the autism-specific word acquisition data were derived from a much larger sample of autistic children than were examined in Haebig et al. and Jiménez et al., which lessen the concern of encountering potential spurious findings. Also, the current study used a word-level approach that allowed for more fine-grained assessment of the association between socialness features and lexical acquisition, while also controlling for important word features that have been found to impact early word learning.

It could have been hypothesized that toddlers may be biased to attend to highly social words because humans are social beings or that highly social words may be more semantically rich and therefore facilitate learning. Though this may pattern be true for word comprehension—which should be pursued in future studies—this is not what we found with our word production data. As Horvath et al. (2018b) suggested, social words may be learned later in vocabulary development because they require children to track the roles of more than one participant (which includes agents, patients, and instruments). This makes the word harder to process. This added relational demand (understanding who is doing what to whom or what object is being manipulated by whom) likely increases the cognitive load and slows down the learning process for young children. Notably, our additional analyses also indicated that our socialness variable was not reducible to argument-structure complexity. This confirms that the Diveica et al. (2023) socialness ratings reflect semantic rather than purely syntactic features.

Furthermore, Diveica et al. (2023) defined socialness more broadly than event participants, with the definition including socialness in terms as social value, social institutions or systems, and social roles. Such a definition would indicate that highly social words may also be more abstract, which tend to be more difficult to learn. Indeed, Diveica et al. (2023) found that an analysis of their full dataset yielded a negative correlation with concreteness (*r* = −0.33). In the current study, socialness and concreteness were negatively correlated, but this association did not reach significance (*p* = 0.14). Notably though, the socialness findings reported in the current study persisted even after controlling for concreteness, as well as word frequency, iconicity, arousal, and valence. Semantically rich words have been noted to facilitate processing in many studies that examine receptive word processing (e.g., lexical decision tasks, Haebig et al., 2015; Nenadić et al., 2024). However, this facilitation pattern often does not hold for word learning studies (e.g., James et al., 2023; Storkel & Adlof, 2009a, 2009b). This inhibitory effect of semantic richness in word learning studies complements the current study’s pattern of findings of highly social words being learned later in vocabulary development, particularly for nouns.

### 4.2. Non-Significant Group Differences and Socialness Effects

We initially expected autistic children to show different patterns in the relationship between socialness and VSoA because of their known social communication differences. Another possibility of a hypothesized interaction between group and socialness ratings could have been that autistic children may be exposed to social words in a different way or frequency relative to non-autistic children, which could possibly impact word learning differences for high vs. low social words. However, in the current study we found no group differences and no interaction between group and socialness. Both autistic and non-autistic children learned social words later. This effect persisted after controlling for other word features that are known to be associated with word learning—frequency, concreteness, iconicity—which therefore highlights the unique contribution of socialness word features in word learning across both groups. Although autistic children often process social information and interactions differently, these differences do not seem to change how socialness features of words are processed by young autistic children relative to non-autistic toddlers. This similarity is consistent with findings from Lin et al. (2022), who reported no group differences in how imageability is related to vocabulary size. The current study aligns with other studies that document similarities in word learning patterns and use of mechanisms that support word learning in autistic and non-autistic children as well. For instance, we know that autistic children benefit from language facilitating strategies like high-quality follow-in commenting and prompts for communication acts (e.g., Clark-Whitney et al., 2022; Haebig et al., 2013; Siller & Sigman, 2008). Additionally, autistic as well as non-autistic learning trajectories align with vocabulary growth models that prioritize the importance of learning from semantic and syntactic structure from the learning environment (e.g., Haebig et al., 2025). Autistic children also attend to syntactic distributions adjacent dependencies to support word learning (e.g., Haebig et al., 2017; Horvath et al., 2018a).

### 4.3. Limitations and Future Directions

This study has several limitations. First, socialness measure we used is based on adult norms (Diveica et al., 2023). Adults conceptualize socialness abstractly (e.g., “trust,” “leader”), whereas children’s semantic representations are often grounded in perceptual and motor experiences (Pruden et al., 2006; Gentner & Boroditsky, 2001). This mismatch may overestimate children’s access to social meanings early in development. Though previous findings have indicated that young children’s early word learning is more heavily influenced by input frequency and perceptible word features like concreteness (Braginsky et al., 2019), it is notable that the current findings revealed that socialness ratings remained a significant variable and explained unique variance in vocabulary acquisition data even after controlling for these other perceptible word features. Furthermore, Diveica et al.’s broad quantification of socialness glosses over subcomponents of social features. Through our extended analyses that included valence and arousal, we found that our socialness measure remained a unique predictor of word acquisition. These results provide a strong argument for considering socialness as a word feature that impacts learning. As such, future work should explore different subcomponents of socialness (e.g., agency, cooperation vs. competition) when examining word learning and word processing in autistic and non-autistic individuals to better understand this effect. Future studies could also explore latent semantic analysis procedures from language samples from autistic and non-autistic children to explore social semantic features.

A second limitation of current study was that socialness ratings were available for only 170 of the 680 words on the CDI, which narrowed the range of words we could examine. This limited dataset restricted the sensitivity to identify potential group differences. Also, our limited sample restricts our ability to explore later lexical development. Though Diveica et al. (2023) developed socialness rating scores for over 8000 English words, currently autism-specific acquisition norms (e.g., age of acquisition or VSoA) for all of the words that have socialness ratings do not exist. The current study served as an initial step to exploring the role of socialness in early vocabulary development in autistic and non-autistic children.

An additional limitation of the current study was that we controlled for word frequency using frequency estimates derived from CHILDES; while this is a rich source to better understand the language environments that children learn within, the children in this corpus are primarily believed to be typically developing. Our attempt to control for input frequency effects could be strengthened if we could include group-specific frequency data. Currently though, a large and public corpus that is specific to autism does not exist (though we know that researchers are working to develop such a resource in the future and have started to share small sets of parent–child transcripts; e.g., ASDBank; MacWhinney & Fromm, 2022). Furthermore, although we controlled for iconicity, concreteness, arousal and valence, other correlated features such as imageability were not included and could explain additional variance. Currently, imageability ratings are not available for all of the CDI words; thus, given that our word socialness ratings also had limited span of the CDI, we did not further restrict our data to also requiring imageability scores. Additionally, our current study focused on semantic features of words, but future work could also examine phonological features of words (e.g., word length, phonotactic probability) in combined models.

Another limitation of the current study was that we relied on group-level acquisition norms rather than data from individual children, which may hide important differences in how children learn words. It is notable though that a child-level approach would not allow for a word-level analysis. Finally, our analyses were limited to English-speaking children, so the patterns we found may not apply to other languages or cultural contexts (e.g., imageability cultural differences; Ma et al., 2009). 

Future research should experimentally examine whether socialness directly affects children’s ability to learn and retain new words. Longitudinal studies could also explore how caregivers adjust the social complexity of their language as children’s vocabulary develops (see Perry et al., 2021 for a similar approach when examining iconicity). Such a study could clarify how caregivers adapt their input over time and identify strategies that support learning socially complex words. Such a study could also examine whether caregivers use social words at different rates when interacting with autistic versus non-autistic children.

## 5. Conclusions

Social word features were linked to later vocabulary acquisition in both autistic and non-autistic children, showing that socially complex words are harder to learn early. This association was strongest for noun acquisition. The current findings highlight an overlap in the word-level factors that shape learning across developmental profiles.

## Figures and Tables

**Table 1:** Multiple Regression Predicting Vocabulary Size of Acquisition (VSoA) From Socialness, Group, and Frequency.

Predictor	B	SE	*t*	*p*
(Intercept)	470.85	36.58	12.87	<0.001
Socialness Ratings	29.95	6.63	4.51	<0.001
Group (Non-autistic vs. Autistic)	9.68	32.88	0.30	0.77
Word Frequency (Log10 CHILDES Freq)	−86.77	10.81	−8.03	<0.001
Socialness Ratings × Group	6.01	9.33	0.64	0.52

*Note.* B = unstandardized regression coefficient; SE = standard error, *F*(4, 334) = 24.79, *p* < 0.001, *R*^2^ = 0.23, adjusted *R*^2^ = 0.22.

**Table 2:** Regression Predicting VSoA From Socialness, Group, Valence, and Arousal.

Predictor	B	SE	*t*	*p*
Intercept	367.27	53.44	6.87	<0.001
Socialness Ratings	24.12	7.28	3.31	<0.001
Group (Non-autistic vs. Autistic)	9.72	35.37	0.28	0.78
Arousal	−1.49	8.15	−0.18	0.86
Valence	−20.59	6.13	−3.36	<0.001
Socialness Ratings × Group	5.99	10.04	0.60	0.55

*Note. N* = 339 (1 case deleted due to missing data). B = unstandardized coefficient; SE = standard error. These patterns of findings remain when word frequency is also included in the model. *F*(5, 333) = 8.26, *p* < 0.001; *R*^2^ = 0.11, adjusted *R*^2^ = 0.10.

**Table 3:** Multiple Regression Predicting VSoA From Socialness and Lexical covariates.

Predictor	B	SE	*t*	*p*
(Intercept)	672.28	60.76	11.06	<0.001
Socialness Ratings	34.63	6.81	5.08	<0.001
Group (Non-autistic vs. Autistic)	6.56	32.73	0.20	0.841
Iconicity Ratings	−2.53	0.63	−4.01	<0.001
Concreteness Ratings	−2.18	1.38	−1.58	0.115
Arousal	1.65	7.81	0.21	0.832
Valence	−20.36	5.73	−3.55	<0.001
Word Frequency	−85.96	12.10	−7.11	<0.001
Socialness Ratings × Group	6.68	9.23	0.72	0.470

*Note*. B = unstandardized coefficient; SE = standard error. *F*(8, 300) = 17.66, *p* < 0.001, *R*^2^ = 0.32, adjusted *R*^2^ = 0.30.

**Table 4:** Final Regression Model Predicting VSoA for Nouns.

Predictor	B	SE	*t*	*p*
(Intercept)	1265.23	98.90	12.80	<0.001
Socialness Ratings	28.52	6.44	4.42	<0.001
Group (Non-autistic vs. Autistic)	12.05	28.31	0.43	0.67
Iconicity Ratings	−0.47	0.66	−0.72	0.48
Concreteness Ratings	−111.20	17.37	−6.42	<0.001
Word Frequency	−184.93	13.50	−13.70	<0.001
Socialness Ratings × Group	4.30	8.51	0.51	0.61

*Note.* B = unstandardized regression coefficient; SE = standard error. *F*(6, 169) = 46.62, *p* < 0.001, *R*^2^ = 0.62 and adjusted *R*^2^ = 0.61. Residual standard error = 86.08. Twenty-eight observations were excluded due to iconicity scores being unavailable for some words.

**Table 5:** Final Regression Model Predicting VSoA for Verbs.

Predictor	B	SE	*t*	*p*
(Intercept)	1015.68	96.24	10.55	<0.001
Socialness Ratings	−4.61	11.71	−0.39	0.70
Group (Non-autistic vs. Autistic)	6.53	65.52	0.10	0.92
Iconicity Ratings	−2.73	0.90	−3.03	0.003
Concreteness Ratings	−79.38	14.41	−5.51	<0.001
Word Frequency	−90.79	17.70	−5.13	<0.001
Socialness Ratings × Group	5.33	16.66	0.32	0.75

*Note.* B = unstandardized regression coefficient; SE = standard error. *F*(6, 72) = 12.65, *p* < 0.001, *R*^2^ = 0.51 and adjusted *R*^2^ = 0.47. Residual standard error = 79.63. One observation was excluded due to missing non-autistic VSoA data.

## Data Availability

We cite the appropriate sources of data that are available publicly or upon reasonable request from the original research team.

## Abbreviations

The following abbreviations are used in this manuscript:

VSoA	Vocabulary Size of Acquisition
AoA	Age of Acquisition
